# Solid-State NMR Spectroscopy: Towards Structural Insights into Starch-Based Materials in the Food Industry

**DOI:** 10.3390/polym14214686

**Published:** 2022-11-02

**Authors:** Mustapha El Hariri El Nokab, Yasser A. Alassmy, Marwan M. Abduljawad, Khalid M. Al-shamrani, Mohammed S. Alnafisah, Zahra Asgar Pour, Chelsea L. Tucker, Khaled O. Sebakhy

**Affiliations:** 1 Zernike Institute for Advanced Materials (ZIAM), University of Groningen, Nijenborgh 4, 9747 AG Groningen, The Netherlands; 2 King Abdulaziz City for Science and Technology (KACST), Riyadh 12354, Saudi Arabia; 3 Engineering and Technology Institute Groningen (ENTEG), University of Groningen, Nijenborgh 4, 9747 AG Groningen, The Netherlands

**Keywords:** solid-state NMR spectroscopy, starch, food science, sustainable polymers, solvent–matrix interactions, sensitivity boosting, polarization enhancement

## Abstract

Solid-state NMR is a nondestructive and noninvasive technique used to study the chemical structure and dynamics of starch-based materials and to bridge the gap between structure–function relationships and industrial applications. The study of crystallinity, chemical modification, product blending, molecular packing, amylose–amylopectin ratio, end chain motion, and solvent–matrix interactions is essential for tailoring starch product properties to various applications. This article aims to provide a comprehensive and critical review of research characterizing starch-based materials using solid-state NMR, and to briefly introduce the most advanced and promising NMR strategies and hardware designs used to overcome the sensitivity and resolution issues involved in structure–function relationships.

## 1. Introduction

The world population is expected to increase to 10 billion by 2050 [1], resulting in growing concerns over global food security. Food production is one of the largest industries globally [2]. Estimates by the United Nations Food and Agriculture Organization (FAO) suggest that a 50% production increase may be needed to meet future demands [1]. A key concern is whether the high demand for food may lead to instability in value-chains, and furthermore, if production targets can be met considering increased water and land scarcity. The future of sustainable food systems may include a shift towards a plant-based diet, as well as significant reductions in food waste [1]. To achieve this, the efficiency of production for existing plant-based foods may need to be improved. Thus, it is critical to understand the connection between the form, function, and properties of our food constituents [3].

Starch is a highly abundant, biodegradable, and hydrophilic carbohydrate typically found in staple crops such as corn, potatoes, wheat, rice, and green fruits [4,5,6,7]. It is an important raw material within a wide range of industries from packaging (e.g., coatings, films adhesives) to biomedical and pharmaceuticals (e.g., tissue and drug carriers) [8]. It is used most frequently in the food industry, where it is estimated that up to 60% [3] of the starch produced is used either as a food product, or a food-based additive for preservative, thickening, texturizing, emulsion stabilization, aroma and flavor encapsulation, or quality enhancement [8,9]. Starch can be used to produce biodegradable packaging films, used to extend the shelf life of foods [10,11], or for encapsulation of food compounds which can improve food quality via protecting bioactive food ingredients from oxidation, or degradation due to UV or acidic conditions [12].

Starch consists of long chains of glucose units connected via glycosidic bonds [7], with a chemical formula of (C_6_H_10_O_5_)_n_. A granule of starch typically contains up to 98-99% (dry weight) of two types of polysaccharide components: amylose and amylopectin [7,8,13,14]. Amylose is a water-soluble, relatively linear [15] polymer made up of glucose units with an α-(1 → 4) glycosidic linkage [8,14,16]. Amylopectin (ca. 60–90% of starch) is a water-insoluble branched polymer of glucose units with multiple short chains linked at α-(1 → 6) to the macromolecule [3]. Amylopectin chains (10 or more glucose units) form double helical structures with either an A-, B- or C- type crystallite. These differ based on unit cell type and packing density. A-type crystallites have a monoclinic unit cell and more compact structure. B-type crystallites have a hexagonal unit cell, an open structure, and a hydrated core [3,17]. The remainder of dry weight of starch consists of a mixture of lipids, minerals, and phosphorus-containing species [3,18].

Starch can be used in a ‘raw’ or ‘modified’ form [5]. The physicochemical and functional properties of raw starch vary for different botanical species [3]. In general, these properties include poor solubility, low shear resistance, low cohesiveness, syneresis, swelling, gelatinization and retrogradation [7,19]. Gelatinization occurs when starch adsorbs water at high temperatures. Under these conditions, starch expands leading to crystalline swelling and an eventual disruption of hydrophobic colloid formation [20]. Retrogradation is a process where upon cooling, starch molecules reorganize and form a viscous, gel-like structure [15,21]. Starch with a high amylose content has a higher tendency to retrograde, due to a low degree of branching which promotes the formation of semi-crystalline structures [3].

**Figure 1:**
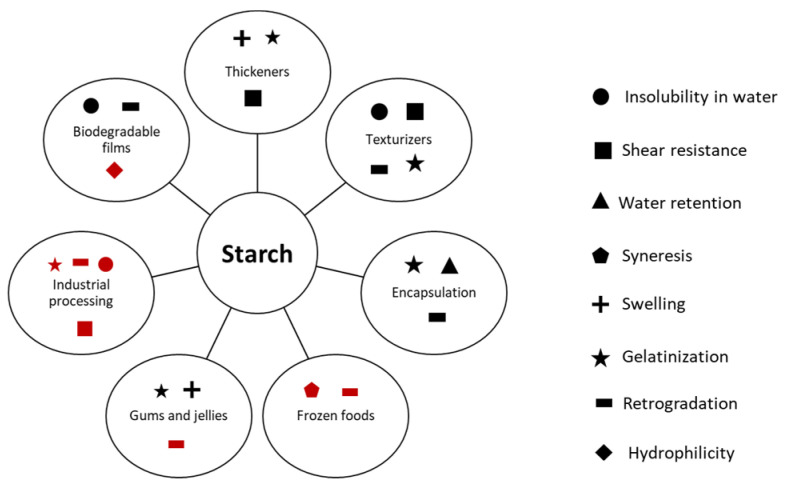
Food-based applications of starch and corresponding properties important for the relative application. Unfavorable characteristics noted in red.

Many of the key properties of starch (insolubility, shear resistance, water retention, gelatinization, retrogradation, hydrophilicity) can be either desirable or unfavorable, depending on the application. Figure 1 shows a selection of food-based applications for starch, noting important properties of starch related to each. In this figure, characteristics unfavorable to the application are shown in red. Swelling and gelatinization, which leads to the formation of a viscous starch paste, is a desirable property for thickeners [3,22] (see Figure 1). However, increased viscosity due to gelatinization and low shear resistance can limit industrial processability. Encapsulation is promoted by starch’s water retention, tailorable viscosity (due to gelatinization), and retrogradation (which slows down release of encapsulated compounds [12,23]). The formation of biodegradable films can also be tailored using retrogradation which can lead to the formation of stronger films [21]. On the other hand, retrogradation can be detrimental for the storage and sensory quality [24] of both fresh and frozen starch-based foods. Other properties like hydrophilicity can be disadvantageous for biodegradable films, which need to have strong barrier properties to prevent migration of film components into food.

To improve raw starch properties, and to tailor these for specific applications, modification is often necessary [7,8,21,25]. Modification can be used to improve processability, physicochemical characteristics but also nutritional quality, texture, and functionalization [4]. Modification can be conducted via chemical, physical, enzymatic, or genetic methods [8].

Chemical modification involves changing the functionalization of the starch either via esterification, etherification, cationization, oxidation or cross-linking [4,7,8]. Physical modification involves the use of heat, moisture, or decomposition. This is typically done to improve water solubility or to reduce particle size [8,21]. Enzymatic modification involves the use of enzymes under mild reaction conditions to change the functionalization of starch with a lower activation energy [4]. This is particularly attractive due to the specificity and selectivity of these treatments, minimizing the formation of unfavorable by-products [4]. Genetic modification involves changing the properties and functionalization of starch by changing the genotype of the crop itself [26]. Biotechnology can improve the starch yield, structure, and functionality (typically termed “molecular farming” [26]).

Due to starch’s important role within food-based industries, its accurate characterization is critical [27]. The techniques used to characterize starch can be segmented broadly into: rheological [28], X-ray techniques [2,17,29,30,31], thermal analysis [32], microscopy [25,33,34,35] and spectroscopic methods including nuclear magnetic resonance (NMR) [36,37,38,39,40], infra-red (IR) spectroscopy [34,41] and Raman spectroscopy [14,31,42,43]. Table 1 shows examples of each of these methods.

Dong et al. [28] (see Table 1) studied the rheological properties of starch nanoparticles. This study showed how starch nanoparticles behave like a viscous liquid at low concentrations and have a gel-like viscosity (with excellent flow behavior) at higher concentrations. This analysis facilitated the optimization of process conditions to achieve desirable processing functionality.

Morphological properties of starch can be analyzed by means of microscopy (see Table 1). In one study [26], scanning electron microscopy (SEM) was used to show how starch’s granule shape can be significantly influenced by plant type. Potato starch was shown to have an oval shape while corn and rice starch was polyhedral in nature. Wheat starch appeared lenticular. Characterization of the shape and size of starch granules is important as these factors influence the gelatinization of starch and its performance as a thickener, texturizer or for encapsulation used (see Figure 1 [19]). Other morphological studies (see Table 1) have shown that SEM can be used to characterize the porous nature of modified starch [25], while TEM can be used to characterize the smoothness of starch after ultrasonication-based modification [35].

X-ray techniques can be used to characterize the structure of starch before and after retrogradation (see Table 1) [2,28,29]. As retrogradation occurs, the amorphous nature of starch changes to a more crystalline state. This can be detrimental or useful, depending on the application, as shown in Figure 1. Retrogradation can be analyzed and quantified using X-ray diffraction (XRD) [30] or via IR and iodine-binding UV [41]. In the former study [30], 2θ angles of a retrograded starch sample from sweet potato were found at 14.7°, 20.7°, 24.3°, 26.5°, and 29.9°. XRD can also be used to distinguish between A- and B-type starch, where strong diffraction patterns can be found either at 15° and 23° 2θ (A-type) or 5.6° and 17° 2θ (B-type) [44].

Thermal techniques such as differential scanning calorimetry (DSC) and thermogravimetric analysis (TGA) are commonly used to determine the glass transition temperature, gelatinization temperature, melting temperature and moisture content of starch and starch composites [32,45,46,47]. One example of this, shown in Table 1, was an analysis of the glass transition temperature (T_g_) and melting point (T_m_) of starch-TiO_2_ nanocomposite films using DSC [32]. In this case, TiO_2_ was shown to increase both T_g_ and T_m_, a beneficial property for packaging systems.

Spectroscopic techniques such as infrared (IR) and Raman spectroscopy (see Table 1) have been used to characterize starch structure and amylose content, respectively [41,42]. These characteristics are important for identifying and predicting retrogradation, which is favored at high amylose–amylopectin ratios and can limit applications of starch (see Figure 1). Solution state nuclear magnetic resonance (NMR) is another spectroscopic technique that can be used to characterize structural features including branching and the degree of substitution for modified starch [39].

In all cases in Table 1, the characterization of starch was needed to explain the connection between form, functionality, and performance of starch for various applications. Recent reviews have focused on the characterization of starch from the perspective of traditionally used methods. Here, the focus is on the characterization of starch using the strong, yet not commonly used technique of solid-state NMR (ssNMR) [48]. As eloquently noted by Blazek et al. [2] “attempts to apply techniques traditionally not widely used in food science provide fascinating challenges and opportunities for modern food materials science”.

ssNMR is a powerful technique, well known for its use in studying the structure and dynamics of carbohydrate polymers [48,49]. This includes, but is not limited to, starch [18,50], cellulose [51] and alginate [49]. While limited in terms of resolution and sensitivity, ssNMR enjoys several advantages over different analytical techniques. ssNMR is a quantitative, non-destructive, and non-invasive experiment. It can be used to obtain information on samples in all different physical states, for both amorphous and heterogeneous compounds [52,53] as well as to measure different nuclei within the same sample.

Highly valuable information can be aggregated when experiments are performed with a combination of different ssNMR techniques—such as magic angle spinning (MAS) and cross polarization (CP) [50]. The CP MAS NMR technique is well known for enhancing the sensitivity of low gyromagnetic ratio nuclei (e.g., ^13^C, ^15^N and ^31^P), via the cross-polarization effect, starting from high gyromagnetic ratio nuclei (e.g., proton) relying on the strong heteronuclear dipolar coupling in the system. However, the technique is not considered quantitative due to its low efficiency when heteronuclear dipolar coupling is weak, as in the case of mobile and highly mobile systems. Single pulse experiments provide quantitative results when set with sufficient recycle delays (five time the spin-lattice relaxation time T1), granting complete relaxation of the targeted nuclei. CP can be considered a useful sensitivity enhancement technique to discriminate rigid molecules while an improved modified version for rigid and mobile molecules exists: the CPSP. CPSP provides its greatest benefits over the standard CP for the ^13^C nuclei with short relaxation values and cross-polarize inefficiently [54]. Different NMR pulse sequences have shown capabilities for detecting mobile regions (regions with weak heteronuclear dipolar coupling) including NOE (Nuclear Overhauser Effect) and INEPT (insensitive nuclei enhanced by polarization transfer). These experiments were able to overcome the efficiency limitations of CP and provided enhanced sensitivity for mobile regions [55].

To obtain deep insights into the carbon skeleton chemical structure for starch-based materials, 2D INADEQUATE (Incredible Natural Abundance Double Quantum Technique) is one of the most powerful NMR techniques available. This technique relies on J(C-C) to provide information about the carbon skeleton. 2D INADEQUATE is not used extensively in characterizing materials due to its extremely low sensitivity when compared to a 1D ^13^C direct excitation experiment.

Enhancement techniques can be used to improve sensitivity of 2D INADEQUATE. For instance, ^13^C isotopic labeling can be used on the sample (e.g., algae, plant and fungal cell wall [48]). This is, however, not used often due to the complexity and cost [48]. Alternatively, a cryogenic probe (cooling the detector coil to 20 K) can be used for polymeric materials, e.g., polyolefins [56]. Enhancement can also be achieved via magic angle spinning dynamic nuclear polarization (MAS-DNP) [48,57].

MAS and CP are standard techniques used in ssNMR for the study of organic and polymeric materials. A wealth of information on carbohydrate polymers can be obtained from these techniques including: polymorphism, degree of substitution/crosslinking, grafting position, crystallinity index, solvent–biopolymer interactions, aggregate formation, polymer chain dynamics and lipid–biopolymer interactions [58,59,60,61].

## 2. Study of Starch Polymorphism

Starch is the most abundant biopolymer in plants, consisting mainly of amylose and amylopectin. These form semicrystalline granules with a wide range of crystallinity between 15 and 45% [62]. Starch’s semicrystalline structure can be classified into A, B and C polymorphs, with the latter existing from the combination of A and B [63]. The crystalline orientation for A and B polymorphs is a parallel double helical strand. They differ in their lattice structure: with the A polymorph having a monoclinic lattice corresponding to a B2 space group with 8 molecules of water, and the B polymorph having a hexagonal lattice corresponding to a P61 space group with 36 molecules of water [62,63]. Another crystalline structure exists, mainly forming after the recrystallization of gelatinized starch, the V-form, and is based on a single helical strand of glucopyranosyl chains [64].

**Figure 2:**
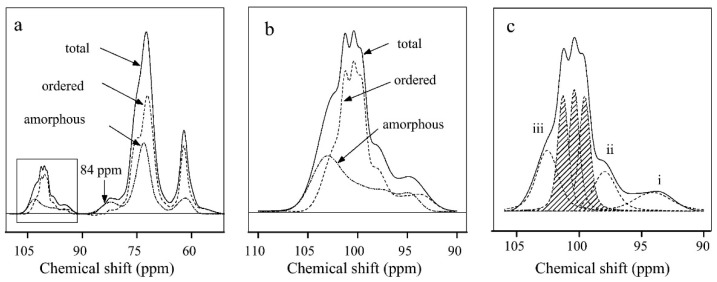
^13^C CP MAS NMR spectrum of recrystallized cassava starch: (**a**) spectrum decomposition into ordered and non-ordered amorphous phases, (**b**) expansion of the C_1_ region between 90 and 110 ppm, and (**c**) deconvolution of the ordered phase in indicating three deconvoluted peaks for A-polymorph, two deconvoluted peaks in the case of B-polymorph and the interfacial phases indicated by i, ii and iii. Adapted with permission from Ref. [43]. Copyright 2012, Elsevier.

The ^13^C CP MAS NMR experiment is a powerful and straightforward analytical tool for studying the molecular organization [65], semicrystalline vs. amorphous structure [66,67,68,69,70], and mobility of the polymeric chains of starch [71,72,73,74]. The spectrum depicted in Figure 2a consists of three main regions including: (I) the region between 60 and 65 ppm, assigned to C_6_, (II) theregion between 68 and 78 ppm, assigned to the ring of the molecule C_2,3_ and _5_, followed by C_4_ at 84 ppm, and (III) the region between 90 and 105 ppm, assigned to C_1_ [43].

The starch polymeric structure is assembled of crystalline and amorphous regions differing in the torsion angles of the α-(1 → 4) glycosidic bond. Thus, carbon atoms at opposite ends of the glycosidic bond possess different local electron densities in their different ordering structures [43]. Upon decomposing and deconvoluting the C1 region in Figure 2b,c, it is possible to determine the type of crystalline packing. Three deconvoluted peaks appear at 99, 100 and 101 ppm, having the same intensity ratio. This indicates three distinct classes of torsion angles were assigned to an A-polymorph with a monoclinic lattice. Two deconvoluted peaks appear at 100 and 101 ppm, which indicates that two distinct classes of torsion angles were assigned to a B-polymorph with a hexagonal lattice. Three different peaks assigned to interfacial conformations were observed at 94, 97 and 103 ppm and represented in Figure 2c. Moreover, the C4 signal at the opposite end of the glycosidic bond at 84 ppm was related directly to the amorphous phase content in the sample, thus showing less information compared to the C1 signal [43,67,68,75].

To obtain higher-resolution spectra, 2D INADEQUATE ssNMR experiments based on NOE (Nuclear Overhauser Effect) and CP were performed on ^13^C-labeled starches produced from *Chlamydomonas reinhardtii* microalgae [76]. The complete assignment of native and retrograded starches (including all the crystalline and amorphous forms) were resolved, and chemical shifts for carbon atoms C2, 3 and 5 (overlapped and poorly resolved in 1D spectra) were fully assigned [76]. The latter have never been reported before.

**Figure 3:**
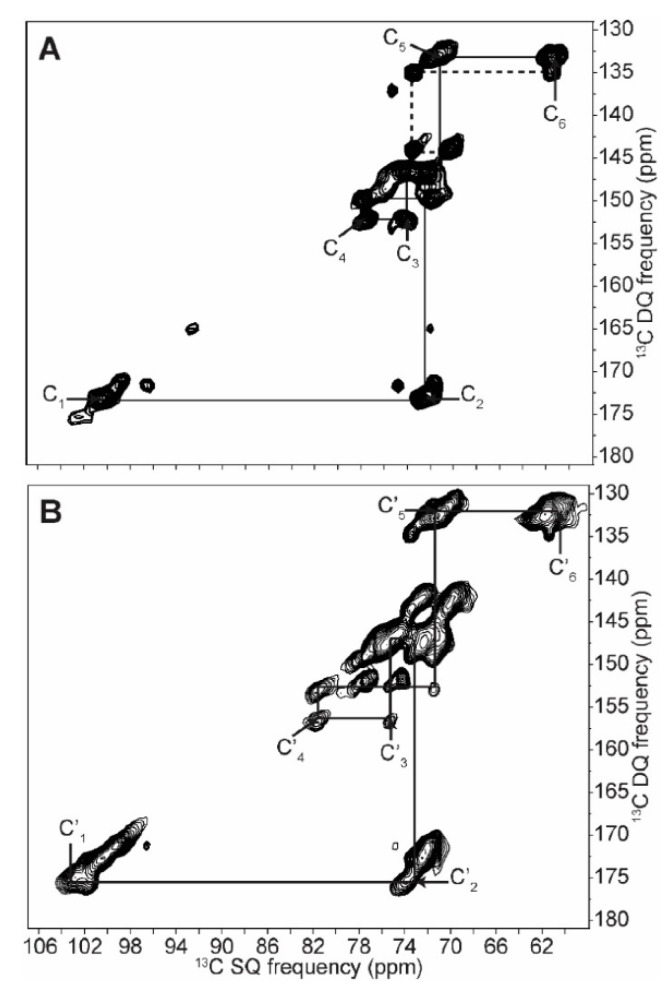
2D INADEQUATE ^13^C ssNMR spectra based on: NOE (**A**) and CP (**B**) for native starch (A-polymorph). Continuous lines indicate the correlation between the crystalline domains represented in (**A**) and the amorphous domains represented in (**B**), while dashed lines indicate end groups. Adapted with permission from Ref. [76]. Copyright 2018, MDPI.

This NOE-based experiment, represented in Figure 3A, was used for signal enhancement of the mobile regions (reported via dashed lines as non-reducing terminal glucose groups) of native starch (A-polymorph). These mobile regions had weak heteronuclear dipolar coupling. Their signal intensities were enhanced due to their close proximity to the directly irradiated nuclei where the NOE is transferred to the mobile region via cross-relaxation effect.

Meanwhile, the CP-based experiment represented in Figure 3B was more efficient for detecting the amorphous regions (high-density populated regions with strong heteronuclear dipolar coupling). This is due to the polarization transfer from high- to low-gyromagnetic-ratio spin-active nuclei (protons to carbon).

The spin correlation between the crystalline domains appeared to be well resolved in the NOE-based experiment, while the amorphous domains were observed better in the CP-based experiment (having different chemical shifts and line shape). Overall, the 2D ssNMR experiments were able to characterize the structure of both highly crystalline amylopectin and poorly crystalline B-type amylose. Moreover, new chemical shifts and multiplicities were assigned and an interpretation for the ordered, disordered, chain length, crystallinity and amylose/amylopectin ratio was provided [76].

## 3. Study of Structural and Dynamic Heterogeneity in Starch

Several factors have a direct effect on, and bear responsibility for, the structure heterogeneity of starch-based products and food products. These factors include: water content [77,78,79,80], pH changes [81,82,83,84], storage conditions [85,86,87,88], temperature and pressure [89,90,91,92] and enzymatic degradation [93,94,95,96].

**Figure 4:**
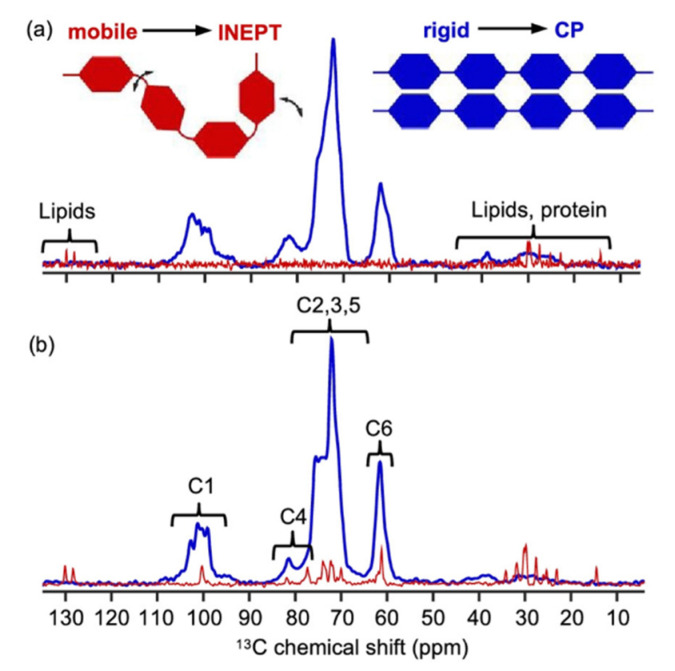
Polarization transfer ^13^C ssNMR spectra comprised of CP and INEPT experiments on: (**a**) dry pasta strand and (**b**) soaked ones. The insert illustrates the mobile (red) and the rigid (blue) domains of the polymeric chain. Adapted with permission from Ref. [97]. Copyright 2021, Wiley.

On a molecular level, these heterogeneities appear in the forms of granule swelling, starch gelatinization and granule disintegration [73,77,97]. However, studying such heterogeneous structures is challenging, with few techniques being capable of holistic analysis. Polarization transfer ^13^C ssNMR spectra based on CP and insensitive nuclei enhanced by polarization transfer (INEPT) experiments are considered an alternative approach. CP-based experiments exhibit better efficiency on rigid crystalline and amorphous structures, while INEPT-based ones depend directly on the mobility of polymeric segments and flexible gelatinized chains [97,98]. The CP spectrum for dry uncooked pasta (represented in Figure 4a) reveals broad peaks of starch’s rigid structure, while INEPT shows no peaks of starch except for some visible peaks related to lipids and proteins. Upon soaking the pasta in water for 1h, the broad peaks in the CP spectrum represented in Figure 4b become narrower, and the C1 region becomes more resolved, showing a mixture of type A and B polymorphic crystalline structures.

The INEPT experiment appears to work better for soaked starch; however, only relatively small peaks were observed from the starch region which could be assigned possibly to mobile fractions of dissolved starch or amylose leakage. The INEPT experiment showed peaks related to lipids and proteins with higher intensities compared to the dry starch pasta [97].

**Figure 5:**
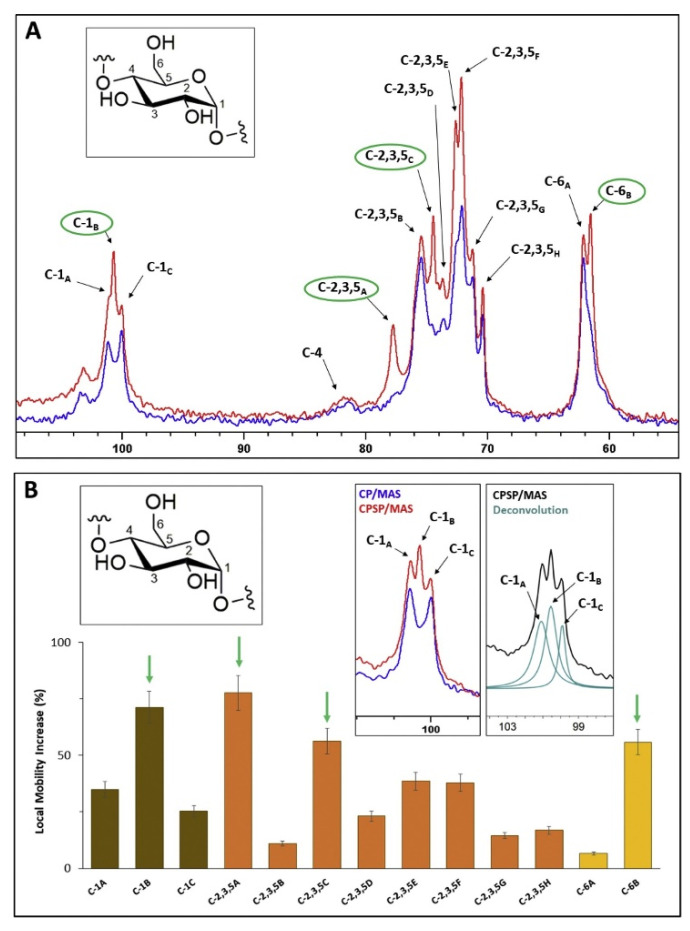
CP and CPSP MAS NMR spectra of normal maize hydrogels: (**A**) an overlay of both spectra with the assignment of peaks with increased local mobility, (**B**) an estimate of the local mobility level for all detected carbon atoms. The insert shows the deconvoluted peak and assignments. Adapted with permission from Ref. [99]. Copyright 2020, Elsevier.

A different approach for measuring and assigning the local mobility of a heterogeneous structured compound is by the comparison of CP and single pulse CP (CPSP) MAS NMR experiments [99]. This approach was used for starch (maize) hydrogels [99]. In this case, the local environments for all carbon atoms were investigated, except for C4 where little difference was observed. Under CPSP conditions, several additional peaks (represented in Figure 5A and labelled in green) were detected when the spectrum was overlaid and compared to the normal CP MAS experiment. These newly detected peaks are considered more mobile compared to the rest of the structure.

To obtain a better understanding, spectral deconvolution was applied to the C1 region, which was separated into three different peaks, represented in the insert of Figure 5B. The side peaks, appearing only in the CP MAS experiment at 99.8 and 100.8 ppm, exhibited reduced local mobility compared to the central peak at 100.3 ppm, appearing exclusively in the CPSP MAS spectrum, which exhibits an increase in the local mobility. The full spectral deconvolution and quantification represented in Figure 5B showed the local mobility and dynamics of the starch hydrogel structure. It also confirmed the change that occurred in the helical packing observed in results from powder X-ray diffraction data, reported in [99].

## 4. Study of Dynamics in Starch in the Presence of Plasticizers and Structural Modifications

Many industrial and food applications of starch are directly related to its physical-chemical properties, such as gelatinization, crystallinity, adhesion, solubility, and viscosity. Starch plasticization, via the addition of water and glycerol in different proportions, can tune the thermoplastic properties and change the phase transition of starch. This, in turn, produces a physically modified starch in a homogeneous polymeric state [100,101,102,103,104].

**Figure 6:**
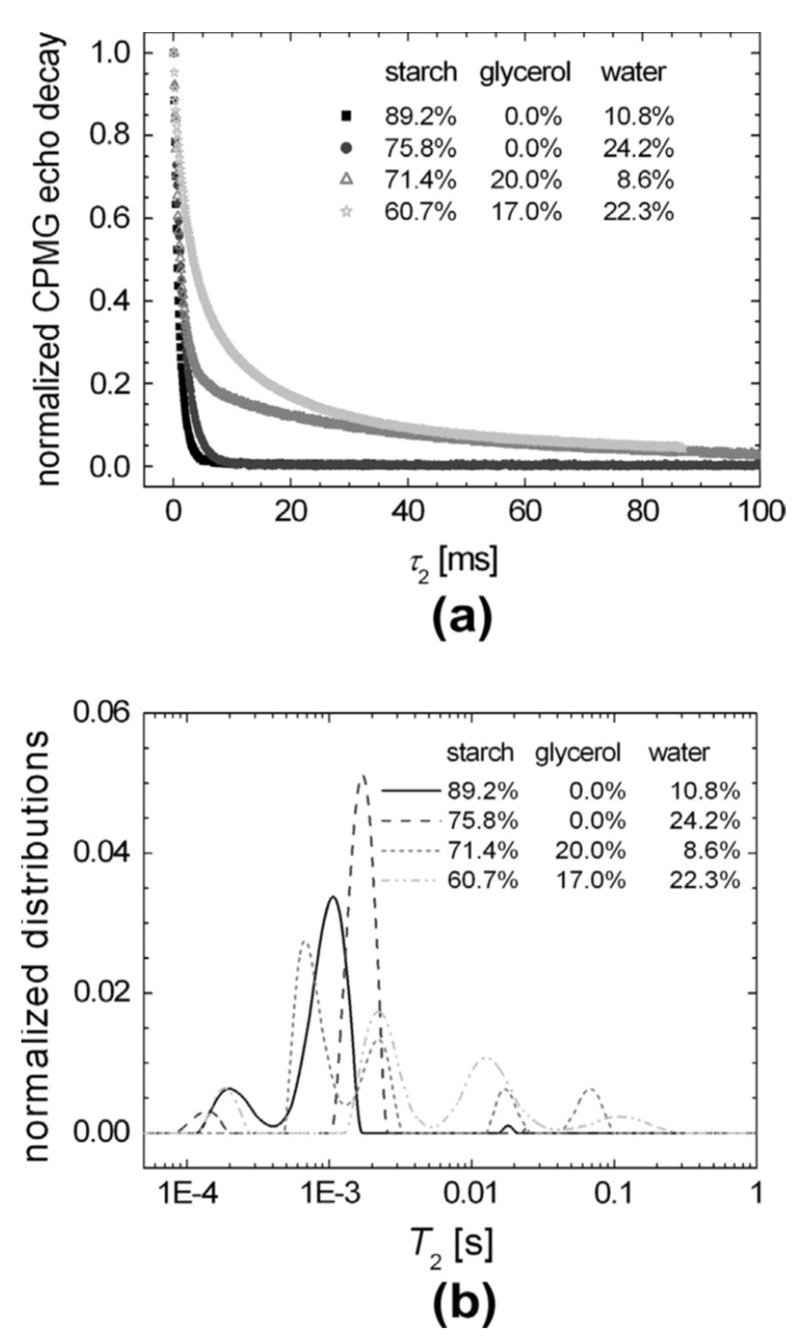
T_2_ relaxation time NMR measurements: (**a**) CPMG-based NMR experiments for starch with variable glycerol and water content; and (**b**) T_2_ time distributions obtained from the CPMG decay curves via Laplace inversion. Adapted with permission from Ref. [107]. Copyright 2013, Elsevier.

Carr–Purcell–Meiboom–Gill (CPMG) echo decay train [105,106] is an essential component in NMR pulse sequences used for measuring the dynamic properties of starch [107]. Train pulses refocus the inhomogeneous broadening of the nuclear spins. This makes it possible to obtain spin–spin T_2_ relaxation decays that possess crucial information regarding the dynamics and composition of native and plasticized starch. The CPMG decay curves presented in Figure 6a show a slight difference for native starch (10.8% water) and starch with addition of water (24.2% water). However, upon the addition of glycerol, a significant difference was revealed. Three separated peaks appeared for native and water mixed starch, as represented in Figure 6b. In contrast, four peaks were observed in the case of glycerol addition (with and without water).

From the three peaks observed for native starch, two could be related to the rigid backbone chain. The first peak at 0.2 msec could be associated with the polymeric chain segments with the lowest mobility located close to the branched backbone. The second peak at 1 msec could be associated with a more mobile segment located away from the core branched region. The third peak at 20 msec represents the mobile branches of the amylopectin microstructure and free amylose end chains.

Upon the addition of water, a shift in the mobile and semi-mobile peaks to higher T_2_ values was observed. The peak corresponding to the rigid component shifted in the opposite direction, thus indicating the formation of soft matter structures. The addition of glycerol lead to the formation of a semi-mobile region. This semi-mobile region consists of two peaks at 0.6 and 2.5 msec and a mobile one at around 20 and 70 msec. This is related to an increase in the amylose free end chains and amylopectin lateral branches. This suggests an increase in the total mobility of the starch polymeric chain [107].

Starch modification and blending with active compounds have been considered a wide sectional area in the food and product industry. However little information, on a molecular level, is known about the dominant interactions and binding sites between the starch and the integrated active compounds [108,109,110,111,112,113,114,115,116]. The interaction between potato starch and cuminaldehyde was analyzed via ssNMR [117], and found to be based on hydrogen bonding, with primary starch binding sites on the oxygen atoms of the hydroxyl-2, 3 functional groups [117].

**Figure 7:**
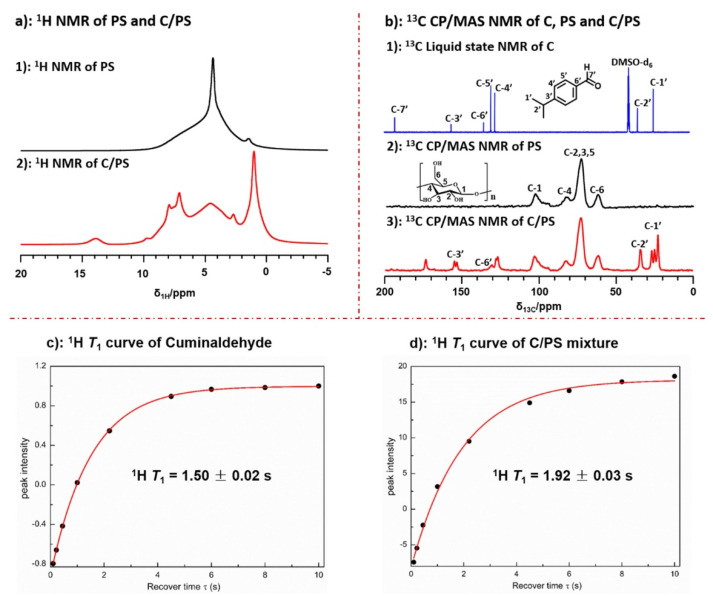
ssNMR techniques for measuring the structure and relaxation properties of PS (black) and C/PS (red): (**a**) single pulse ^1^H MAS NMR spectra for PS and C/PS; (**b**) ^13^C CP MAS NMR spectra for PS and C/PS compared to solution state spectrum of cuminaldehyde; and (**c**) ^1^H T_1_ relaxation curve for cuminaldehyde; and (**d**) ^1^H T_1_ relaxation curve for C/PS. Adapted with permission from Ref. [117]. Copyright 2022, Elsevier.

Conventional solid-state NMR experiments, including the ^1^H single pulse depicted in Figure 7a and the ^13^C CP MAS experiments presented in Figure 7b, were performed on porous starch (PS) and a blend of starch and cuminaldehyde (C/PS). For the purposes of comparison, a ^13^C solution state NMR experiment was conducted on cuminaldehyde (C).

The results obtained from proton and carbon solid-state NMR spectra were consistent with starch loaded with significant amounts of cuminaldehyde. Upon comparing the solution state spectra of cuminaldehyde to the solid-state one for C/PS, it was found that only a single methyl peak appeared in the solution state spectrum, while three peaks between 20 and 30 ppm were detected in the solid-state spectrum. This indicates interactions between cuminaldehyde methyl groups and starch. It also indicates that three different environments of free and adsorbed cuminaldehyde were present. The same phenomena appeared in C_3_ of the phenyl group, where two different peaks appeared in the solid-state spectrum compared to one single peak in solution state.

Molecular mobility was investigated by measuring the relaxation properties for cuminaldehyde represented in Figure 7c and C/PS represented in Figure 7d. The ^1^H T_1_ measurements show a significant difference in the molecular dynamics between cuminaldehyde in its pure form (^1^H T_1_ = 1.5 sec) and when adsorbed in the starch structure (^1^H T_1_ = 1.92 sec). The increase in the ^1^H T_1_ values for cuminaldehyde was attributed to the restricted mobility of the cuminaldehyde molecules in the starch structure, thus resulting in an increase in the longitudinal relaxation [117].

## 5. Future Perspectives and Conclusions

Starch is one of the most abundant components in food products. However, it possesses a complicated semicrystalline packing ordered structure, which creates a dilemma for investigators resolving the molecular structure, polymorphism and solvent–matrix interactions. Several analytical techniques have been used to investigate the structure of starch, but none as efficient at providing information about the structure and dynamics in a quantitative, non-destructive way. ssNMR has proven to be a useful technique when it comes to the inspection of the structure and dynamics of food derivatives, since the majority of such compounds have disordered to semicrystalline structures with wide range of polymorphs. Using ssNMR, valuable information can be gathered about the dynamics, crystallinity, water pools, degree of modification, starch blending and structural heterogeneity of starch-based compounds.

Recently, ssNMR has seen major developments in pulse sequences such as water-edited 1D ^13^C and 2D ^13^C-^13^C CP MAS experiments and 3D NMR experiments. It has also seen a wide range of newly developed hardware including, but not limited to, Pulse Field Gradient NMR, comprehensive multiphase NMR, low field NMR, magnetic resonance imaging and microimaging, ultra-high-magnetic-field magnets, CryoProbes, and ultra-fast MAS probes. This opens the way for resolving the full structure of native, gelatinized, plasticized and modified starch, as well as starch-based products.

Advanced hyperpolarization and sensitivity boosting techniques have developed significantly and have been applied to different materials, including carbohydrate and cellulose-based systems [48,57,118,119]. This is especially true for MAS-DNP, where this technique enabled the performance of low sensitivity and time-consuming experiments. These advances enhance conventional NMR techniques [120], and provide higher sensitivity and resolution. This enables investigations to reach a deeper level of understanding with regards to starch structure, reaction mechanisms, intermediates detection, starch-blends interactions, ion diffusion and drug delivery release performance.

## Figures and Tables

**Table 1:** Characterization methods for native and modified starch and starch-based biodegradable materials.

Broad Technique	Analytical Method	Property Analyzed	Description	Reference
Rheology	Rheometer	Viscosity	Continuous shear tests performed on starch nanoparticles to measure apparent viscosity	[28]
Microscopy	Scanning electron microscope(SEM)	Granule morphology	SEM morphology comparison between potato, corn, wheat, and rice as well as enzymatically modified starches	[25,33]
	Transmission electron microscopy (TEM)	Granule shape and surface features	Ultrasonically treated (modified) starch analyzed in thin cross-sections of granules obtained by ultramicrotome	[35]
	Atomic force microscopy (AFM)	Morphology of films	Starch-based biodegradable film surfaces analyzed by AFM in tapping model	[33]
X-ray technique	Small angle neutron scattering (SANS)	Lamellar structure	Lamellar architecture and crystalline structures of starch during hydrolysis	[2]
	Small angle X-ray scattering (SANS)	Nanostructure	Nanostructure of the freeze-dried wheat starch pastes after repeated heating and cooling	[29]
	X-ray diffraction	Crystallite morphology	X-ray diffraction patterns of sweet potato amylose before and after retrogradation using copper, nickel foil-filtered and Ka radiation	[30]
Thermal analysis	Differential scanning calorimetry (DSC)	Glass transition temperature and melting point	Starch-TiO_2_ nanocomposite films glass transition temperature and melting point analysis by DSC	[32]
Spectroscopic	Nuclear magnetic resonance (NMR)	Structural features	Characterization of native and modified starch and starch gelatinization procedure	[39,40]
	Infra-red (IR) spectroscopy	Structural features	Analysis of the structure of retrograded maize starch	[41]
	Raman spectroscopy	Amylose content	Determination of amylose content in starch FT-Raman spectroscopy with germanium detector	[42]
